# Arterial Network Geometric Characteristics and Regulation of Capillary Blood Flow in Hamster Skeletal Muscle Microcirculation

**DOI:** 10.3389/fphys.2018.01953

**Published:** 2019-01-18

**Authors:** Dominga Lapi, Martina Di Maro, Teresa Mastantuono, Noemy Starita, Mauro Ursino, Antonio Colantuoni

**Affiliations:** ^1^Department of Clinical Medicine and Surgery, Medical School, Federico II University, Naples, Italy; ^2^Molecular Biology and Viral Oncology Unit, Istituto Nazionale Tumori Fondazione G. Pascale (IRCCS), Naples, Italy; ^3^Department of Electrical, Electronic and Information Engineering, University of Bologna, Bologna, Italy

**Keywords:** skeletal muscle microcirculation, Strahler's ordering scheme, arteriolar rhythmic diameter changes, capillary perfusion, blood flow regulation, L-arginine, papaverine

## Abstract

This study was aimed to characterize the geometric arrangement of hamster skeletal muscle arteriolar networks and to assess the *in vivo* rhythmic diameter changes of arterioles to clarify regulatory mechanisms of the capillary perfusion. The experimental study was carried out in male Syrian hamsters implanted with a plastic chamber in the dorsum skin under pentobarbital anesthesia. The skeletal muscle microvessels were visualized by fluorescence microscopy. The vessel diameters, lengths and the rhythmic diameter changes of arterioles were analyzed with computer-assisted techniques. The arterioles were classified according to a centripetal ordering scheme. In hamster skeletal muscle microvasculature the terminal branchings, differentiated in long and short terminal arteriolar trees (TATs), originated from anastomotic vessels, defined “arcading” arterioles. The long TATs presented different frequencies along the branching vessels; order 4 arterioles had frequencies lower than those observed in the order 3, 2, and 1 vessels. The short TAT order 3 arterioles, directly originating from “arcading” parent vessels, showed a frequency dominating all daughter arterioles. The amplitude of diameter variations in larger vessels was in the range 30–40% of mean diameter, while it was 80–100% in order 3, 2, and 1 vessels. Therefore, the complete constriction of arterioles, caused an intermittent capillary blood perfusion. L-arginine or papaverine infusion caused dilation of arterioles and transient disappearing of vasomotion waves and induced perfusion of all capillaries spreading from short and long TAT arrangements. Therefore, the capillary blood flow was modulated by changes in diameter of terminal arterioles penetrating within the skeletal muscle fibers, facilitating redistribution of blood flow according to the metabolic demands of tissues.

## Introduction

Arterioles display spontaneous rhythmic diameter changes, previously called vasomotion. The accompanying variations in resistance result in capillary blood flow oscillations. Vasomotion was explained as a mechanism for increasing arteriolar flow conductance (= 1/resistance), which plays an important role in controlling blood pressure (Nicoll and Webb, 1955; Zweifach, 1971; Slaaf et al., 1988; Aalkjær et al., 2011) and has been demonstrated to increase oxygen supply to tissue under conditions of low oxygenation (hypoxia) (Zweifach, 1971). The rationale is that vessel conductance is proportional to the fourth power of its diameter (Poiseuille's law). As a result, the contribution to the average conductance during the diameter increase phase is greater than during diameter decrease, leading to net conductance increase (Aalkjær et al., 2011).

Several mechanisms are known to be involved in this phenomenon, including autonomic nervous discharge, circulating substances, mechanical stimulation of vessels, myogenic or shear-dependent or metabolic or conducted responses propagated along the vessels.

However, the physiological role and underlying mechanisms of vasomotion are not fully understood (Aalkjær et al., 2011). After the fundamental study by Nicoll and Webb (1955), Colantuoni et al. (1984, 1985) reported arteriolar vasomotion in hamster skin fold window preparation, characterized by rhythmic oscillations in vessel diameters. Bouskela and Grampp (1992) showed in the hamster cheek pouch by intravital microscopy that the arteriolar vasomotion was regularly present in healthy preparations, independent of anesthesia or combined nervous alpha- and beta-adrenergic receptor blockade. In arterioles with internal diameters between 13 and 52 microns, the vasomotion frequency (3–15 cycles/min) and amplitude (2–10 microns) were not significantly correlated to the vessel size. These oscillations of the arteriolar lumen diameter were able to modify blood flow in the corresponding capillary networks.

The regulation of capillary blood flow was suggested by Krogh in his pioneering studies, introducing the capillary recruitment mechanism: each capillary dominated by a muscle precapillary sphincter. Under resting conditions 10–20% of capillaries in a skeletal muscle microvascular network are perfused, while the remaining are recruited according to the muscle cell metabolic demand under stimulation or during activity (Krogh, 1922). A lot of data have been reported after Krogh's hypothesis on capillary perfusion regulation. However, the issue is still under question: under resting conditions a percentage of capillaries are perfused and the mechanism of recruitment is still unclear (Duling and Weiner, 1972; Intaglietta and Tompkins, 1973; Lindbom and Arfors, 1985; Bertuglia et al., 1991; Schmidt et al., 1993; Parthasarathi and Lipowsky, 1999). Drenckhahn and Weigelt suggested that the capillary blood flow is regulated by the capillary contractility; while Schmid-Schönbein proposed a model based on plugging of capillaries by formed elements of the blood (Schmid-Schönbein, 1987; Delashaw and Duling, 1988). A study, carried out by Delashaw and Duling, described the microcirculatory anatomy of the hamster tibialis anterior muscle showing that terminal arteriole supplies two microvascular units or a “unit pair,” consisting of group of 12–20 of capillaries which run parallel to muscle fibers. The units are drained by a common terminal venule. Under basal conditions all capillaries were perfused, although the velocities in individual vessels were often different. The Authors studied the response to increase in oxygen pressure, muscle contraction, and phenylephrine superfusion. They found that topical phenylephrine was able to induce simultaneous arrest of capillary flow of a unit pair in 18 of 21 units pairs (Delashaw and Duling, 1988). However, the crucial point is still to clarify the role of terminal arteriolar networks; the differential behavior of arterioles and capillaries is not easy to reconcile with the anatomy of the microcirculation in different tissues, because the mechanism of capillary recruitment has been unclear (Fry et al., 2013). Fry et al., indeed, have studied capillary recruitment in a theoretical model for metabolic blood flow regulation in a heterogeneous network, based on experimental data from hamster cremaster muscle. They suggest that capillary recruitment can occur as a consequence of local regulation of arteriolar tone and the resulting non-uniform changes in red blood cell fluxes. Therefore, it is of interest to correlate the capillary flow to the arteriolar rhythmic diameter changes. This study was aimed to assess the *in vivo* geometric features of terminal arteriolar networks in the hamster dorsal cutaneous muscle and to characterize the rhythmic diameter changes of arterioles, focusing our attention on capillary functional recruitment under baseline conditions and during nitric oxide-dependent or independent arteriolar dilation.

## Methods

Eleven male Syrian Golden hamsters (Charles River, Calco, Italy) weighing 80–100 g were subjected to implantation of the chamber in the dorsal skinfold as previously reported (Colantuoni et al., 1985); seven of these animals were treated with L-arginine or papaverine (10 and 0.3 mg/100 g b.w. intravenously infused, respectively). In brief, the animal was anesthetized (5 mg/kg body wt, pentobarbital i.p.); then, two symmetrical plastic-frames were implanted into a dorsal skinfold of hamsters. A round area of the dorsum skin and the underlying skin muscle (15 mm diameter) was removed from one side of the symmetrical fold exposing the opposite layer of skin muscle (muscle cutaneous maximus), anatomically attached to the subcutaneous tissue. A microcover glass covered the tissue, fixed to one of the plastic frames, while the other part remained open. Heparinized catheters were inserted in the jugular vein and in the carotid artery to inject fluorescent tracers and to measure systemic arterial blood pressure, respectively. Catheters, passing under the skin to the neck, were fixed to the window. The animals recovered for 48 h in an incubator at 30 ± 0.5°C (Colantuoni et al., 1984).

All experiments conform to the *Guide for the Care and Use of Laboratory Animals* published by the US National Institutes of Health (NIH Publication No. 85-23, revised 1996) and to institutional rules for the care and handling of experimental animals. The protocol was approved by the “Federico II” University of Naples Ethical Committee (Protocol No. 3685/13/CB).

The observation of skeletal muscle microvasculature was performed on unanesthetized animals constrained in a tube, without impeding respiration; the extending frame of the chamber was fixed to the microscopic stage. Trans-illumination and epi-illumination were used for microscopic observation; a 100 W tungsten halogen lamp was utilized with a round heat-absorbing filter and a 414 nm bandpass filter. A Leitz Orthoplan microscope was equipped with a long-working distance objective (× 4, NA 0.12; × 20, NA 0.25; × 32, NA 0.60) and × 10 eyepiece (Colantuoni et al., 1984).

The microvasculature was investigated by fluorescence microscopy, i.v. injecting fluorescein isothiocyanate bound to dextran (mol wt 150 kDa; 50 mg/100 g body wt in 5% solution). Vessel networks were then televised by a DAGE MTI 300 low light-level camera, connected to a Sony PVM 122 CE monitor and to a computer for recording by imaging computerized-system. Fluorescence images were recorded with a Leitz I_2_ Ploemopack filter block. Arterial blood pressure and heart rate were recorded by a Gould Windograph, through a Statham PD 23 transducer connected to the catheterized artery. The chamber temperature was maintained at 30 ± 0.5°C by warmed air.

The diameter and length of vessels were evaluated by a computerized method (MIP Image; Institute of Clinical Physiology, CNR, Pisa, Italy); moreover, the vessel diameter was measured with an additional method or the shearing method (Intaglietta and Tompkins) (IPM shearing monitor 109, San Diego, CA, USA) (Intaglietta and Tompkins, 1973).

To avoid bias due to single operator judgement, measurements by two blind operators were compared: the results overlapped in all cases. The velocity of the red blood cells was measured by dual window velocimeter (102 B IPM) and by stop-frame images (Colantuoni et al., 1984, 1990). Moreover, for each terminal arteriolar tree (TAT) we measured the capillary red blood cell velocity (RBC) in each capillary by computer-assisted method (frame by frame) and we evaluated the blood flow (Q) according to the following equation: Q = V × A, where V was the RBC (mm/s) and A was the cross-sectional area.

The time and frequency domains of the diameter changes were assessed by power spectrum analysis, utilizing Fourier transform method. In particular, the Fourier transform was based on the generalized short time Fourier transform (GSTFT) (Varanini et al., 1998; Pradhan and Chakravarthy, 2011; Varanini, 2011), a multiresolution transform which allowed us to choose, at each frequency, the most appropriate balance between time and frequency resolution according to the user's requirements. “A Hamming window was used and spectra were computed at frequencies spaced proportionally to the frequency resolution. The power density spectral distribution was obtained by time averaging the time-frequency power density representation. This technique permits to evaluate non-stationary data, such as those represented by rhythmic variations in vessel diameters” (Rossi et al., 2005; Lapi et al., 2017).

We utilized 2 min recordings, while digitation rate was 2 Hz. The computational system yielded the spectral components of the diameter time series; moreover, the absolute, fractional, and normalized values of the corresponding spectral power were evaluated in the frequency domain.

The arteriolar networks were characterized using low magnification photographs of the chamber. The arterioles were recorded with both trans-illumination and epi-illumination; then, the anatomical arrangement of each network was reconstructed from playbacks and stop-frame images.

In each animal the vessels were characterized and assigned order according to the Strahler's scheme, as previously described (Lapi et al., 2008). Briefly, we classified the terminal branchings of arteriolar networks originating from larger arterioles feeding the muscle. At first, the capillaries were identified and assigned order 0. Thereafter, the arterioles originating the capillaries were assigned order 1 and these upstream were attributed progressively higher orders, as previously reported (Lapi et al., 2008). When two vessels of the same order joined, the parent vessel was assigned the next highest order, while retained the higher of the two orders in the case of different orders in daughter vessels. We used the Strahler's method to assign order numbers to vessels of each order. The mean and standard deviation (SD) of the vessel diameter of an arbitrary order n were named D_n_ and Δ_n_, respectively. Thus, we defined a range of diameters around D_n_ and defined a vessel of order n, when its diameter was between these bounds:

(1) $$\begin{array}{r} \left[\left(D_{\text{n}-1}\;+\;\Delta_{\text{n}-1}\right)\;+\;\left(D_{\text{n}}-\Delta_{\text{n}}\right)\right]/2 \end{array}$$

on the left and

(2) $$\begin{array}{r} \left[\left(D_{\text{n}}\;+\;\Delta_{\text{n}}\right)\;+\;\left(D_{\text{n}+1}\;+\;\Delta_{\text{n}+1}\right)\right]/2 \end{array}$$

on the right. The final result was a system where the vessel diameter ranges of successive orders did not present overlap. In hamster skeletal muscle as well as in rat pial (Lapi et al., 2008) and in pig coronary microvascular systems, each blood vessel between two nodes of bifurcation is called a segment (Kassab et al., 1993). In the Strahler's ordering scheme segments are connected in series so that they can be considered as a single tube, called element.

The ratio of the overall vessel segments (S) to the overall vessel elements (E) in any given order describe the S/E ratio, which was calculated. This value is crucial to characterize symmetry or asymmetry of microvascular bifurcations and, sequentially, distribution of blood flow in the microvasculature.

We observed that vessels of order n could spring from vessels of orders *n* + 1, *n* + 2, …. Therefore, we were able to implement a mathematical model reported as a “connectivity matrix,” the component of which in row *n* and column *m* was the ratio of the overall elements of order *n* originating from elements in order *m*, as previously reported (Kassab et al., 1993; Lapi et al., 2008). To experimentally obtain the matrix for skeletal muscle microcirculation, we grouped all vascular branches into elements, then recorded for each element of order *m* the number of elements of orders *m, m*-1, *m*-2, …., that arose directly from that element. Finally, we calculated the mean value and SD of each component of the matrix. Previously, the connectivity matrix was utilized to determine the mathematical models of pig coronary and rat pial microcirculation (Kassab et al., 1993; Lapi et al., 2008).

All values were reported as means ± SEM; for connectivity matrix values, we calculated means ± SD. A statistical package, SPSS/PC+ 14.00 was utilized for statistical significance analysis. Normal distribution was evaluated with the Kolmogorov-Smirnov test. According to data distribution we used parametric (Student's test, ANOVA and Sheffé *post-hoc* test) or non-parametric tests (Mann-Whitney and Kruskal-Wallis tests); with parametric tests we compared bifurcation numbers among experimental groups, with non-parametric tests diameter and length data. Statistical significance was set at *p* < 0.05.

## Results

**Figure 1:**
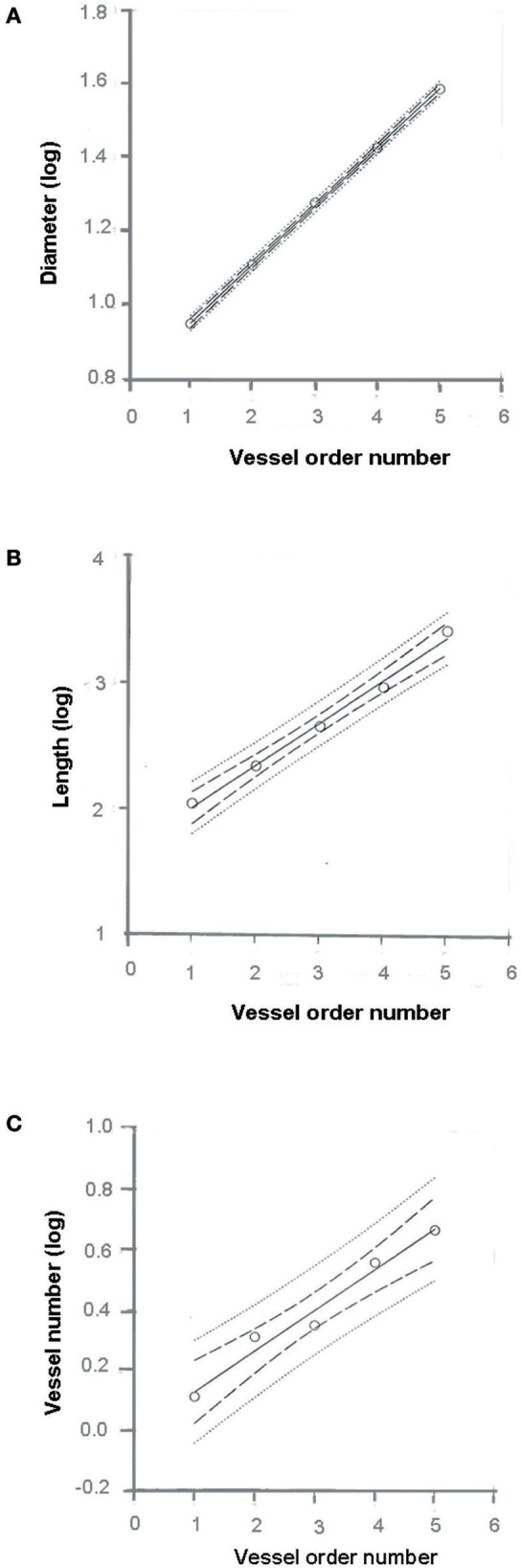
Relationships between mean diameter logarithm **(A)**, length logarithm **(B)**, logarithm of vessel element number in successive orders of vessels **(C)**, and arteriolar order number. The multiple lines indicate confidence intervals.

The hamster skeletal muscle microcirculation showed arterio-arteriolar anastomoses originating the terminal branchings. These anastomotic vessels corresponded to the large arterioles called “arcading” in different experimental models. The terminal branchings were characterized by arterioles penetrating into the muscle fibers and giving origin to the capillaries. Two or more capillaries branched from the most distal arterioles and ran predominantly parallel to the muscle fibers. Order 0 was assigned to the capillaries, according to Strahler's method; thereafter, the terminal arterioles were assigned order 1 and the vessels upstream were assigned progressively higher orders: the anastomotic arterioles were assigned order 5. In this experimental model, all terminal branchings originated from arcading arterioles giving origin to order 4 or 3 arterioles (Table 1). Pooling all data obtained from 11 hamsters, the diameter distribution in successive orders of arterioles obeyed Horton's law, according to the following equation:

(3) $$\begin{array}{r} Log_{10}\;D_{\text{n}}=a\;+b_{\text{n}}, \end{array}$$

where a and b are two constants. According to the least/squares method, we calculated the empirical constants: a = 0.789 and b = 0.161, respectively. The diameter logarithm was directly proportional to vessel order number (Figure 1A and Table 2). The ratio diameter, evaluated from the slope of the curve, was 1.45.

**Table 1:** Diameter and length of each arteriolar order under baseline conditions.

**Order**	**Arterioles *N***	**Diameter (μm)**	**Length (μm)**	**Hamsters *N***
1	507	9.0 ± 0.7^*^	110 ± 24	11
2	172	12.9 ± 1.5^*^	219 ± 61	11
3	62	19.4 ± 3.0^*^	467 ± 114	11
4	16	26.9 ± 2.6^*^	949 ± 144	11
5	11	39.6 ± 5.7^*^	2,689 ± 999	11

* *P < 0.01 vs. different order. The diameters represent the average maximum diameter during 2 min of vasomotion cycles*.

**Table 2:** Empirical constants a and b of equations 1, 2, and 3 for semilogarithmic relationships between mean diameter, length, number of vessel elements, and order number of arterioles.

	**Equation**		
	**(1) Diameter log_**10**_ Dn = a + bn**	**(2) Length log_**10**_ Ln = a + bn**	**(3) Arteriolar number log_**10**_ Nn = a + bn**
A	0.789	1.690	3.097
B	0.161	0.328	−0.435
*R*^2^	0.931	0.919	0.979
Ratio	1.45	2.12	2.72

*Date derived from 11 hamsters were used. Ratio is that between successive orders. R^2^ = correlation coefficient*.

The vessel length changed in successive orders of arterioles according to Horton's law:

(4) $$\begin{array}{r} Log_{10}\;L_{\text{n}}=a\;+b_{\text{n}}, \end{array}$$

where a and b were 1.690 and 0.328, respectively. The ratio length was 2.12 (Figure 1B and Table 2).

The last parameter dependent on vessel order was the branching number. The relationship between the branching number logarithm and the vessel order number, according to Horton's law, was:

(5) $$\begin{array}{r} Log_{10}\;N_{\text{n}}=a\;+b_{\text{n}}, \end{array}$$

where a = 3.097 and b = −0.435; the ratio branching was 2.72 (Figure 1C and Table 2).

**Figure 2:**
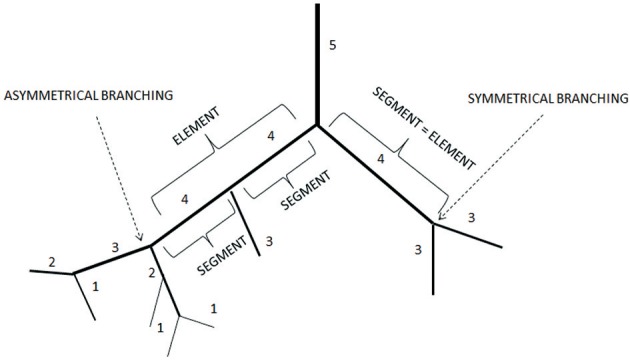
Element/Segment ratio in the arteriolar networks in the hamster skin fold window preparation. When one element was constituted by one segment the bifurcation was symmetrical. When one element was constituted by two or more segments the bifurcation was asymmetrical.

Moreover, we calculated the segment/element ratio to have information on symmetry o asymmetry of bifurcations in the skeletal muscle microcirculation assuming that the ratio equal to 1 indicated complete symmetry, ratios >1 bifurcation asymmetry (Figure 2 and Table 3).

**Table 3:** S/E in each order of vessels.

**Order**	**S/E**	***N***
1	1.31	640
2	2.07	340
3	2.28	130
4	3.66	55
5	4.72	51

*Values are means ± SD. N, Number of vessels observed for each order. S/E is the ratio of the total number of segments in a given order to the total number of elements in that order. This ratio is also the average number of vessel segments in series for each order of vessels*.

The branching vessels in the networks were described in detail by the connectivity matrix, showing the connections of blood vessels of one order to another. In Table 4 it is possible to observe that order 5 arterioles gave origin to 4 order 5 vessels (0.45 × 10), 17 order 4 vessels (1.72 × 10), 14 order 3 vessels (1.45 × 10), and 1 order 2 vessels (0.09 × 10). No vessel of order 1 or 0 originated from order 5 arterioles. Moreover, several order 3 and 2 vessels derived from order 4 parent arterioles. Order 3 vessels gave origin to most order 2 arterioles, while order 2 vessels gave origin to most order 1 arterioles. Finally, order 1 vessels gave origin to the capillaries (Table 4).

**Table 4:** Connectivity matrix.

**Order *n***	**Order** ***m***				
	**1**	**2**	**3**	**4**	**5**
0	2.26 ± 0.23	0.11 ± 0.19	0	0	0
1	0.02 ± 0.05	3.11 ± 0.77	0.72 ± 1.02	0.17 ± 0.41	0
2	0	0.05 ± 0.16	2.60 ± 0.81	1.83 ± 2.32	0.09 ± 0.30
3	0	0	0.09 ± 0.22	2.17 ± 1.60	1.45 ± 1.37
4	0	0	0	0	1.73 ± 1.55
5	0	0	0	0	0.45 ± 0.68

*Values are means ± SD. An element (m, n) in row m and column n is the ratio of the total number of elements of order m that spring directly from parent elements of order n divided by the total number of elements*.

**Figure 3:**
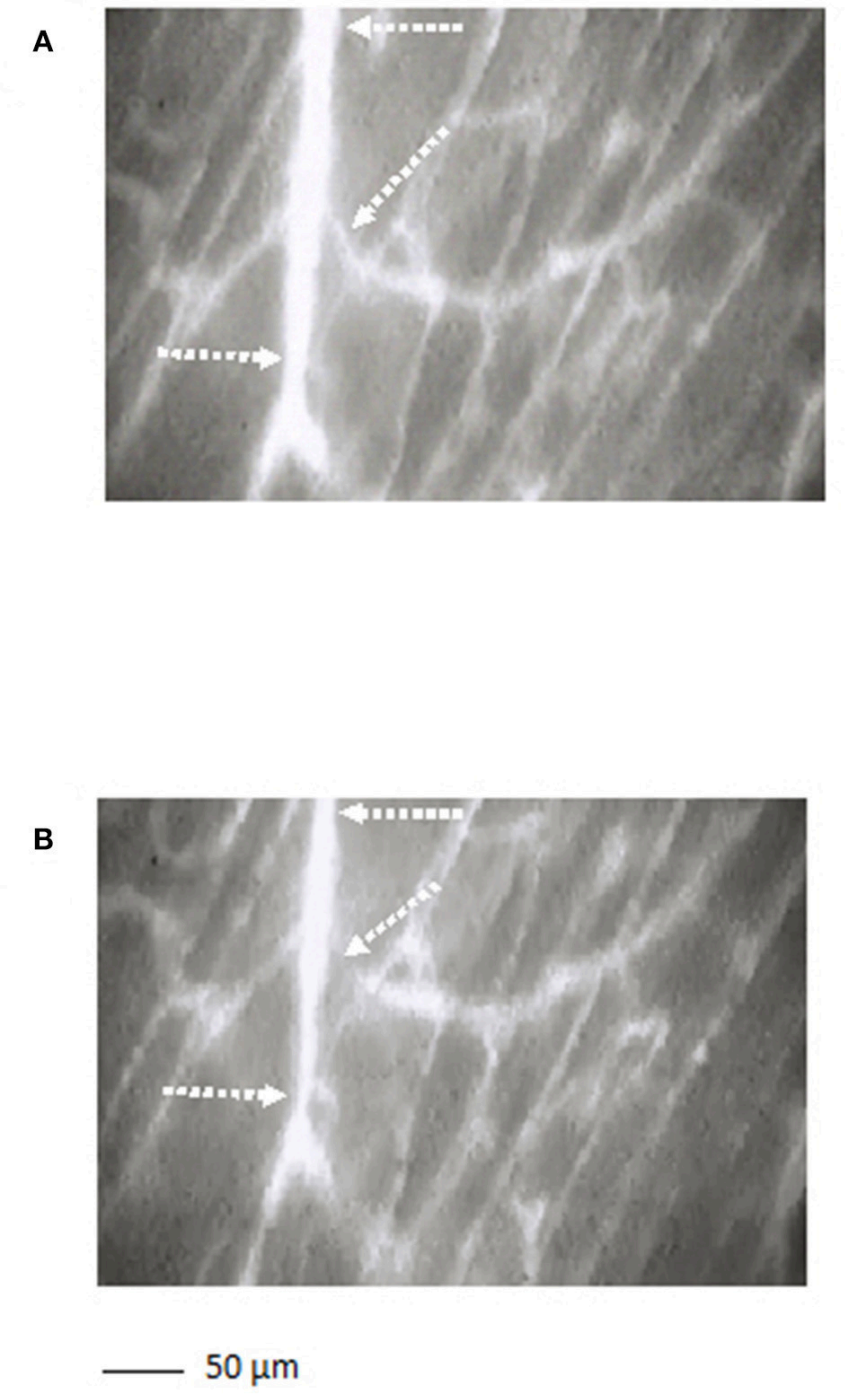
A long terminal arteriolar tree order 4 arteriole, spreading from arcading parent vessel, undergoing vasomotion [**(A)** dilation indicated by the dotted arrows; **(B)** constriction indicated by the dotted arrows]. Scale bar: ___ 50 μm.

**Figure 4:**
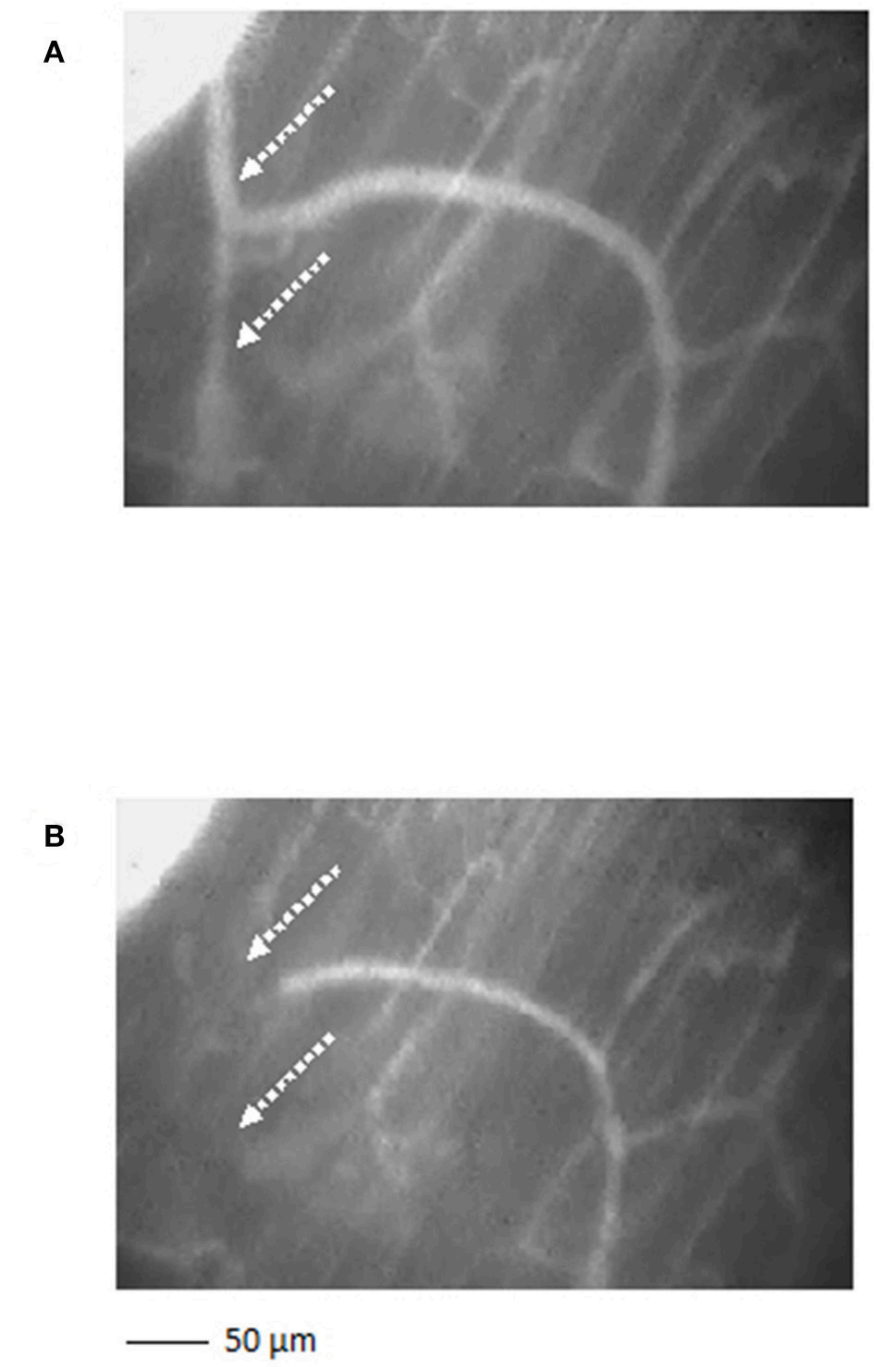
A short terminal arteriolar tree order 3 arteriole, spreading from arcading parent vessel, undergoing vasomotion [**(A)** dilation indicated by the dotted arrows; **(B)** constriction indicated by the dotted arrows]. Scale bar: ___ 50 μm.

The arterioles originating from order 5 vessels, denoted as terminal branchings, were differentiated into long and short terminal arteriolar trees (TATs). The long TATs presented order 4 arterioles branching from arcading arterioles (Figure 3), while the short TATs order 3 arterioles (Figure 4). The daughter vessels, therefore, had significant different diameters when comparing long and short TATs. In all experimental preparations the arterioles showed rhythmic diameter changes; terminal branching vessels presented oscillations in diameter with contraction and relaxation waves originating at the bifurcations from arcading arterioles. The structural arrangement in long and short TATs resulted in significantly different vasomotion frequencies.

**Figure 5:**
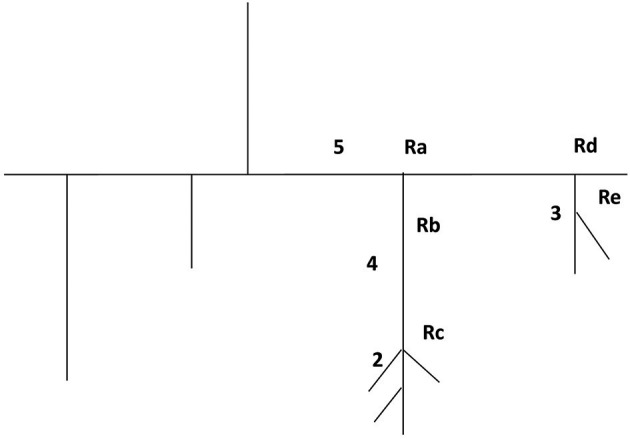
Schematic illustration of a microvascular network with the five recording points (Ra, Rb, Rc, Rd, and Re).

**Figure 6:**
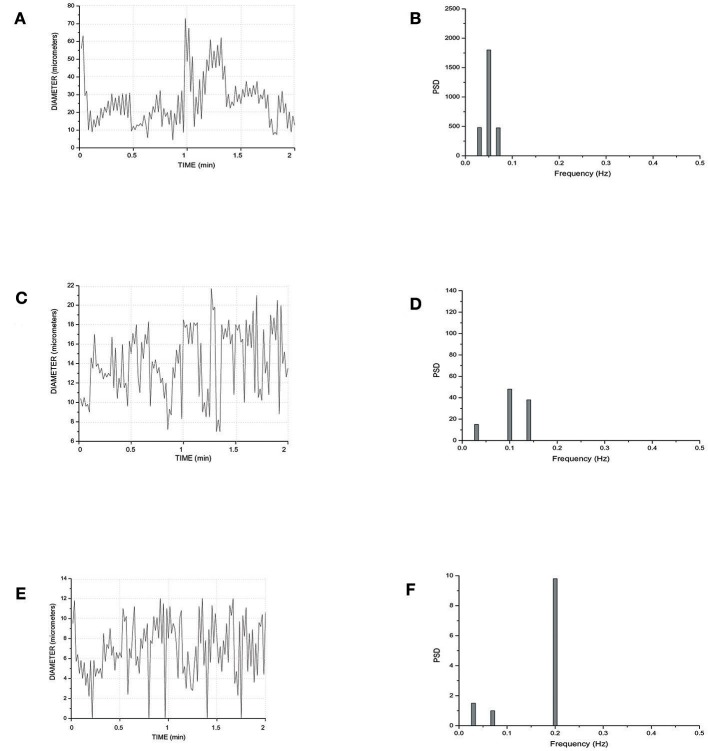
Rhythmic diameter changes of an order 5 arteriole of a long terminal arteriolar tree **(A)** recorded in Ra (Figure 4) and the corresponding power spectrum **(B)**. Rhythmic diameter changes of an order 4 arteriole of a long terminal arteriolar tree **(C)** recorded in Rb (Figure 4) and the corresponding power spectrum **(D)**. Rhythmic diameter changes of an order 2 arteriole of a long terminal arteriolar tree **(E)** recorded in Rc (Figure 4) and the corresponding power spectrum **(F)**. PSD, Power Spectrum Density (μm^2^/Hz).

Figure 5 reports the scheme of a typical microvascular network (long and short TATs) with the vasomotion recording points, while Figure 6 shows the arteriolar rhythmic diameter changes and the corresponding power spectral density. The arcading vessel presented rhythmic changes in diameter with a fundamental frequency of 0.05 ± 0.01 Hz (3 cycles/min) (Figures 6A,B). Order 4 arteriole, initial vessel in the long TATs, had a frequency of 0.10 ± 0.02 Hz (6 cycles/min) (Figures 6C,D). Along the order 4 arteriole, reported in the scheme, there was a branching order 2 vessel with the highest frequency of diameter oscillations (0.22 ± 0.03 Hz, 13.2 cycles/min) (Figures 6E,F). The diameter change amplitude progressively increased from arcading vessel (30–40% of mean diameter) to order 4 arteriole (50–70% of mean diameter) up to order 2 vessel (90–100% of mean diameter). The smallest order 2 completely constricted, obliterating the lumen and inducing stop and go blood flow in the downstream capillaries during a complete vasomotion cycle.

**Figure 7:**
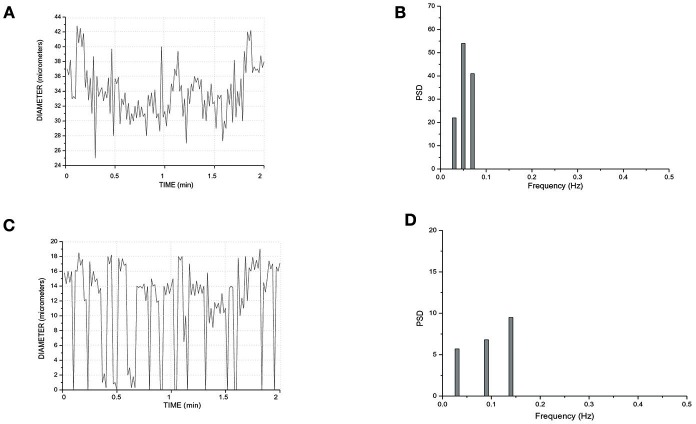
Rhythmic diameter changes of an order 5 arteriole of a short terminal arteriolar tree **(A)** recorded in Rd (Figure 4) and the corresponding power spectrum **(B)**. Rhythmic diameter changes of an order 3 arteriole of a short terminal arteriolar tree **(C)** recorded in Re (Figure 4) and the corresponding power spectrum **(D)**. PSD, Power Spectrum Density (μm^2^/Hz).

In a short TAT typical network, the fundamental frequency of the arcading arteriole did not change compared to that detected in the long TATs (Figures 7A,B). Order 3 vessel directly springing from arcading arteriole showed a frequency of 0.14 ± 0.04 Hz (8.4 cycles/min) (Figures 7C,D). This vessel constricted to the extent able to obliterate the lumen during a vasomotion cycle (Video S1). Moreover, the vasomotion waves originating from order 3 arteriole bifurcation spread along all daughter vessels causing the blood flow to cease in all capillaries. Therefore, the short TATs presented the same fundamental frequency dominating all downstream vessels: capillaries were perfused during arteriolar dilation and resulted not perfused during constriction; consequently, the capillary perfusion was regulated by order 3 vessel activity.

Pooling all experimental data, vasomotion fundamental frequency of order 5 arterioles was significantly different compared with those evaluated in order 4, 3, 2, and 1 arterioles. The order 4 or 3 arterioles (belonging to long TATs) showed slightly lower frequencies compared to those detected in short TAT order 3 vessels. Conversely, order 2 and 1 arterioles springing from order 4 vessels (long TATs) presented higher frequencies compared with those detected in the short TAT arterioles (Table 5).

**Table 5:** Frequency **(A)** and amplitude, expressed as percentage of the average diameter **(B)**.

**Order**	**Long TATs**	**Short TATs**
**A**		
5	0.01–0.08	
4	0.08–0.12	
3	0.09–0.20	0.08–0.16
2	0.11–0.25	0.08–0.16
1	0.11–0.25	0.08–0.16
**B**		
5	30–40	
4	50–70	
3	80–100	80–100
2	80–100	80–100
1	80–100	80–100

It is interesting to note that order 5 vasomotion fundamental wave was transmitted to the branching vessels. Therefore, the power spectrum analysis of order 4 vessel rhythmic diameter changes detected the same frequency component transmitted from order 5 parent arterioles. Therefore, there was a complex superposition of waves in the terminal networks.

The amplitude of vasomotion waves changed comparing terminal long and short TATs. In long TAT networks, order 4 and 3 vessels did not completely obliterate the lumen; therefore, their per cent amplitude was 50 ± 10 and 70 ± 10 of the mean diameter. Order 2 and 1 arterioles presented percent amplitude up to 100% of the mean diameter. Generally, the oscillation amplitude in the smaller vessels was affected by the vasomotion waves originating from order 2 and 1 bifurcation sites. Downstream order 1 vessels showed overlapping frequencies and the same amplitude compared to order 2 arterioles.

In the short TAT networks, the oscillation amplitudes were affected by order 3 vasomotion waves spreading to downstream vessels. Consequently, the per cent amplitude was 80–100 in order 3, 2, and 1 arterioles. The lumen obliteration of order 3 arterioles and branching vessels caused the capillary blood flow to stop: the capillary perfusion resulted intermittent.

In the terminal networks, the blood was flowing from arterioles to capillaries; however, in arcading arterioles the blood flow changed frequently in direction and velocity. Finally, the long and short TAT networks gave origin to a different number of capillaries, 45 ± 6 and 24 ± 9, respectively (*n* = 340 short TAT capillaries; *n* = 750 long TAT capillaries). The different TAT microarchitecture was accompanied by diversified RBC in capillaries arising from long or short TATs. Capillaries springing from the short TATs represented a functional unit with synchronization of the blood flow; such harmonization derived from order 3 arteriolar vasomotion. In the long TATs the capillary blood flow did not depend on order 4 arteriole activity, but the blood supply was regulated by summation of order 4, 3, 2, and 1 arteriole waves of dilation and constriction, when present.

We measured the RBC velocity in the capillaries of long or short TATs and evaluated the blood flow for each TAT. We observed that in the short TAT capillaries, the average of RBC was 0.11 ± 0.02 mm/s with calculated blood flow of 72 ± 6 nl/s, during a vasomotion cycle, taking into account an average capillary diameter of 5.9 ± 0.2 μm and an average capillary number of 24 ± 9. We detected synchronized blood flow in all capillaries, due to closing and opening of supplying arterioles. In long TATs, the blood flow was synchronous in most capillaries, with different capillary RBC in 1/3rd of capillaries. We measured the RBC velocity in each capillary of long TATs and calculated the blood flow during vasomotion cycles. Taking into account an average capillary diameter of 5.8 ± 0.3 μm and an average RBC velocity of 0.11 ± 0.02 mm/s, for 30 ± 3 capillaries and an average diameter of 6.2 ± 0.2 μm for 15 ± 3 capillaries with an average RBC velocity of 0.17 ± 0.02 mm/s, the calculated blood flow was 156.15 ± 3.05 nl/s.

**Figure 8:**
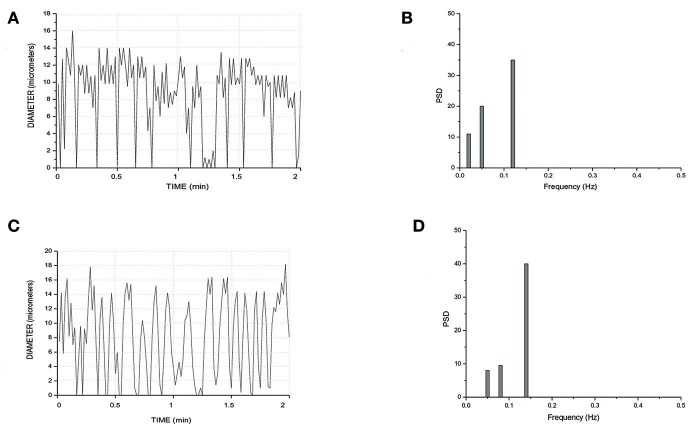
Rhythmic diameter changes of an order 3 arteriole in baseline conditions **(A)** and the corresponding power spectrum **(B)**. Rhythmic diameter changes after L-arginine administration **(C)** and the corresponding power spectrum **(D)**. PSD, Power Spectrum Density (μm^2^/Hz).

Finally, all arteriolar orders of hamsters, administered with i.v. L-arginine, showed a biphasic response: within the first minutes of infusion (4 ± 1 min) the vessels dilated with disappearing of vasomotion waves. Within 5 ± 2 min of stop infusion, there was a recovery of rhythmic diameter changes showing increase in their amplitude: order 2 arteriole maximum diameter during vasomotion cycle was 20.0 ± 1.5 μm; however, there were no significant changes in frequency, even though the trend was toward an increase (Figure 8). The same data were detected with papaverine i.v. administration: all order arterioles first dilated with consequent disappearing of the vasomotion activity; few minutes after the end of the infusion the vessels recovered the rhythmic diameter oscillations showing increase in their amplitude (data not shown). However, the arteriolar dilation induced simultaneous perfusion of all capillaries, in both short and long TATs, where there was perfusion synchronization of all capillaries. During dilation of all arteriolar vessels and vasomotion disappearing, there was an increase in RBC velocity in both long and short TAT capillaries: there was synchronization of all perfused capillaries and the RBC velocity was in the same range for both short and long TAT capillaries: 0.30 ± 0.3 mm/s for all capillaries (*n* = 210 short and *n* = 350 long TAT networks). We measured RBC velocity in capillaries of short and long TATs. We observed that in the same capillaries studied under baseline vasomotion cycles, the dilation induced an increase in blood flow. In short TATs, we calculated blood flow of 196.8 ± 11.5 nl/s in the same 24 ± 9 capillaries above reported, with an average RBC of 0.30 ± 0.02 mm/s; on the other hand in long TAT capillaries we found an increase of blood flow up to 373.35 ± 12.40 nl/s with an average RBC velocity of 0.30 ± 0.02 mm/s. The percentage increases were by 273 ± 9% or by 239 ± 6% of baseline values for the short or long TATs, respectively.

## Discussion

The results of the present study indicate that the hamster skeletal muscle microcirculation was characterized by anastomotic arteriolar networks, organized in “arcading” systems. These arcade arterioles originated daughter vessels spreading among the muscle fibers, according to the terminal patterns: the long and short TATs, presenting different diameter and length of the arterial blood vessels up to the capillaries.

We differentiated the arterioles by diameter, length and branching according to a centripetal ordering scheme (Strahler's method). The geometric characteristics of each arteriolar order demonstrate that diameter, length, and bifurcation increased by a constant according to the order number, as expected by Horton's law, in a system defined “fractal.” In a fractal distribution, indeed, diameters, lengths and bifurcations increase with the increase in arteriolar orders. The constant was evaluated by the slope of regression curve between logarithm of diameter or length or bifurcation and arteriolar order. Our results are in agreement with those previously reported (Kassab et al., 1993; Lapi et al., 2008).

Moreover, we estimated the segments/elements ratio and implemented a connectivity matrix to define the asymmetry of the arteriolar bifurcations. The segments/elements (S/E) ratio demonstrates that the vessels were connected in series, an arrangement useful to describe an electrical circuit fitting the skeletal muscle system. The bifurcation asymmetry must be taken into account for the distribution of microvascular blood flow: the most asymmetrical vessels were order 5 and 4 arterioles, where the ratios were as high as 4.72 and 3.66, respectively, indicating that those vessels play the major role in distributing blood to smaller vessels.

We implemented the connectivity matrix, suggested as “a tool for calculating pressure, flow and vascular volume in coronary networks” (Kassab et al., 1993) and successively in rat pial networks (Lapi et al., 2008). Our data may be useful in calculating these parameters also for skeletal muscle microcirculation. The matrix allowed us to define the number of daughter vessels spreading from parent vessels: order 5 arterioles gave origin to several vessels, but no order 1 or 0 vessels originated from these order 5 arterioles. Moreover, many order 3 and 2 vessels derived from order 4 arterioles. Order 3 vessels gave origin to most order 2 arterioles with few order 1 or 3 vessels. Order 2 arterioles originated several order 1 vessels and few capillaries, while order 1 arterioles gave origin to capillaries. The connectivity relationship may be important to estimate blood flow in skeletal muscle hemodynamic studies and to characterize the functional properties of microvessels, as reported in previous studies (Kassab et al., 1993; Lapi et al., 2008).

It is worth noting that these geometric features characterize the arteriolar terminal branchings independently of the studied tissue. Kassab studied the morphometric characteristics of pig coronary arteries and reported overlapping data (Kassab et al., 1993); moreover, Lapi et al. demonstrated the vessel distribution is fractal in the rat pial microvasculature (Lapi et al., 2008).

It is interesting to point out that the skeletal muscle terminal arterioles were characterized by rhythmic diameter changes regulating the blood flow redistribution into microcirculation. From sequential registration of vasomotion waves it is of evidence that the dilation and constriction activities began at branching of vessels spreading from arcading arterioles. In particular, the terminal branchings denoted a different behavior according to the terminal arteriolar tree's length.

The short TATs constituted a system efficiently regulated: order 3 arterioles could constrict so as to obliterate the vascular lumen stopping blood flow in all downstream vessels. Therefore, the capillary blood flow was intermittent and presented a different velocity when compared with capillaries originating from long TATs, showing blood flow regulated by order 3 and 2 or 1 arteriole activities.

Therefore, it is possible to hypothesize that in the skeletal muscle microcirculation arcading arterioles represented a blood storage, able to facilitate the blood flow redistribution to the fibers by opening of the terminal branchings. These vessels were equipped with a switch on and off time-dependent system that allowed blood to reach or not the level of muscle tissue cells. The closing and opening activity was likely due to the characteristics of the smooth muscle cells endowed in the vessel walls, branching from the arcading arterioles (Delashaw and Duling, 1988; Colantuoni and Bertuglia, 1997; Nilsson and Aalkjaer, 2003; Haddock and Hill, 2005). On these cells influences were exerted by myogenic, neuronal and metabolic activities. The capillary deriving from terminal arterioles had different hemodynamic characteristics depending on parent vessel length. Therefore, the tissue perfusion was regulated by vasomotion arising and spreading along the terminal microcirculation.

The arteriolar rhythmic diameter changes are due to vascular smooth muscle cells that at the beginning of terminal loops show long lasting Ca^++^ channels (Bova et al., 1990; Iino et al., 1994; Miriel et al., 1999; Ruehlmann et al., 2000). These cells operate as peripheral pacemakers able to regulate the blood flow supplying the muscle fibers. Contradictory results were reported in a previous study on the “random” generation of the arteriolar vasomotion in hamster cheek pouch (Bouskela and Grampp, 1992). In hamster skeletal muscle the arteriolar rhythmic diameter changes appeared to explicate the main rule in the blood flow regulation, representing the physiological site of peripheral resistance control.

In the present study the vasomotion waves were analyzed by power spectrum analysis which identified the frequency components of the diameter oscillations. Microvascular networks showed arteriolar vasomotion with different fundamental frequencies (i.e., the frequency with the highest amplitude) and percentage amplitudes. The larger arterioles presented lower frequencies of activity compared with those in smaller vessels: the short TAT order 3 arterioles had frequencies in the range 0.10–0.18 Hz dominating all daughter arterioles (order 2 and 1).

The long TATs had different frequencies along the network; order 4 arterioles, originating from arcading arterioles, had frequencies lower (0.08–0.12 Hz) than those observed in the order 3, and 2 or 1 smaller vessels: these vessels showed frequencies in the ranges 0.09–0.20 Hz (order 3) and 0.11–0.25 Hz, respectively.

The amplitude of diameter variations in order 5 vessels was in the range 30–40% of mean diameter, while it was 80–100% in order 3, 2, and 1 vessels (short TATs). Therefore, the complete arteriolar constriction caused blood flow to stop in capillaries with consequent intermittent blood flow. We assume that the dynamic myogenic response was predominant in smaller arterioles, whereas it progressively decreased in upstream parent vessels. These observations are in accord with previous studies demonstrating that the sympathetic nervous system innervation was sparse in smaller vessels (Furness and Marshall, 1974). Therefore, starting from arcading arterioles up to the terminal arterioles, the local metabolic factors were likely to dominate in the smaller vessels.

It is important to note that long TATs showed a higher number of capillaries compared with those observed in short TATs; moreover, the capillaries originated from long TATs showed asynchrony in blood flow correlated to vasomotion in order 3 or 2 or 1 vessels. Capillaries derived from short TATs presented highly synchronized blood flow. The different perfusion pattern caused varying RBC due to the vascular arrangement and consequent resistance in the two terminal loops: in short TATs the capillary RBC was different compared with those in long TATs.

Previously, Delashaw and Duling studying another experimental preparation (hamster tibialis anterior muscle) found that terminal arteriole supplies two microvascular units or unit pairs. They stimulated the microcirculation by oxygen pressure increase, muscle contraction and phenylephrine superfusion and demonstrated derecruitment or arrest of blood flow perfusion by increase in oxygen pressure or phenylephrine superfusion, in most of investigated networks. In this model with different microvascular organization compared with the cutaneous muscle one, however, there were no data on spontaneous activity of arterioles, but metabolic or pharmacological stimulations were able to affect blood flow regulation of capillaries (Delashaw and Duling, 1988).

In hamster cutaneous muscle microvasculature the main regulation of capillary perfusion appeared related to the arteriolar rhythmic diameter changes, with no clear evidence of “precapillary sphincters,” as previously reported (Delashaw and Duling, 1988). It is worth noting that L-arginine or papaverine, known to induce NO-dependent and NO-independent vasodilation, respectively, induced increase in diameter of arterioles and consequent decrease in diameter oscillations, as shown in different models (Lapi et al., 2017). However, disappearing of waves was transient, because there was diameter oscillation recovery within few minutes of stop-injection. NO is known to interfere with Ca^++^ fluxes in the vascular muscle cells (Ruehlmann et al., 2000), while papaverine has been suggested to induce muscle relaxation without NO implication, through activation of phosphodiesterase and reduction of myosin phosphorylation (Hocking et al., 2016). However, the recovery of vasomotion was characterized by higher amplitude of waves, because the maximum diameter, attained during vasomotion cycles, was 20 μm (on the average for order 2 arterioles). In recent years, vasomotion has been correlated to cellular oscillators, differentiated into cytosolic or membrane oscillators (Nilsson and Aalkjaer, 2003; Haddock and Hill, 2005). The cytosolic one is characterized by the release of Ca^++^ from intracellular stores, such as sarcoplasmic reticulum, causing Ca^++^ waves. These rhythmic waves are not synchronized between neighboring cells (Peng et al., 2001; Aalkjaer and Nilsson, 2005). Membrane oscillators play a major role for intercellular synchronization in the vessel wall. The global [Ca^++^] oscillations are determined by the voltage-dependent mechanism present in the synchronized vascular smooth muscle cells, where rhythmic potentials cause the rhythmic influx of Ca^++^ through voltage-dependent Ca^++^ channels.

Our data indicate that arteriolar dilation induced by L-arginine or papaverine could be related to the interference on Ca^++^ fluxes in vascular muscle cells causing an increase in RBC and flow in all capillaries of short TAT networks. Interestingly, arteriolar dilation in long TATs caused an increase in blood flow in all capillaries with synchronization of perfusion. Therefore, our data indicate that recruitment of capillaries could be related to different mechanisms: the first represented by vasomotion disappearing and sustained dilation of arterioles feeding the capillaries: under these conditions the flow increase was related to the decrease in contraction time of feeding arterioles (short TATs). The second mechanisms consisted in the recruitment of all capillaries spreading from long TAT arterioles, with perfusion synchronization and marked increase in RBC velocity, compared to baseline conditions. Therefore, the recruitment could markedly increase blood flow in skeletal muscle microcirculation. We calculated an increase of blood flow by 273 ± 9% and by 239 ± 6% of baseline values for the short and long TAT networks, respectively, during vasodilation induced by L-arginine or papaverine.

In conclusion, the hamster cutaneous muscle microcirculation originated from anastomotic arteriole networks, functioning as peripheral blood reservoirs, able to supply blood flow to the muscle fibers through terminal branchings. Our data clearly show that the nitric oxide participates actively to the modulation of vascular tone, likely balancing the vasoconstrictor discharge of the sympathetic nervous system and all substances able to induce vasoconstriction. In conclusion, the waves were the results of myogenic activity influenced by different parameters likely related to endothelial cell and autonomic nervous activity.

## Author Contributions

DL: ideation of experiments and text writing; MD: data elaboration; TM: elaboration results; NS: elaboration manuscript; MU: processing data; AC: ideation of experiments, elaboration data, and revision of the text.

### Conflict of Interest Statement

The authors declare that the research was conducted in the absence of any commercial or financial relationships that could be construed as a potential conflict of interest.

## Supplementary Material

Video S1 Real-time recording of arteriolar rhythmic diameter changes in a short TAT.
